# Communicating human biomonitoring results to ensure policy coherence with public health recommendations: analysing breastmilk whilst protecting, promoting and supporting breastfeeding

**DOI:** 10.1186/1476-069X-7-S1-S6

**Published:** 2008-06-05

**Authors:** Maryse Arendt

**Affiliations:** 1 Institute for Improvements around Birth of Initiativ Liewensufank, 20 rue de Contern, 5955 Itzig, Luxembourg

## Abstract

This article addresses the problem of how to ensure consistency in messages communicating public health recommendations on environmental health and on child health. The World Health Organization states that the protection, promotion and support of breastfeeding rank among the most effective interventions to improve child survival. International public health policy recommends exclusive breastfeeding for six months, followed by continued breastfeeding with the addition of safe and adequate complementary foods for two years and beyond. Biomonitoring of breastmilk is used as an indicator of environmental pollution ending up in mankind. This article will therefore present the biomonitoring results of concentrations of residues in breastmilk in a wider context. These results are the mirror that reflects the chemical substances accumulated in the bodies of both men and women in the course of a lifetime. The accumulated substances in our bodies may have an effect on male or female reproductive cells; they are present in the womb, directly affecting the environment of the fragile developing foetus; they are also present in breastmilk. Evidence of man-made chemical residues in breastmilk can provide a shock tactic to push for stronger laws to protect the environment. However, messages about chemicals detected in breastmilk can become dramatized by the media and cause a backlash against breastfeeding, thus contradicting the public health messages issued by the World Health Organization. Analyses of breastmilk show the presence of important nutritional components and live protective factors active in building up the immune system, in gastro intestinal maturation, in immune defence and in providing antiviral, antiparasitic and antibacterial activity. Through cohort studies researchers in environmental health have concluded that long-term breastfeeding counterbalances the effect of prenatal exposure to chemicals causing delay in mental and psychomotor development. Therefore caution should be exercised when presenting the results of biomonitoring of breastmilk. The results should be a motivation to enact strong legislation on chemicals and review the use of chemical substances present in breastmilk, but the results should not be used to undermine the confidence in breastmilk as the optimal food for infants and young children.

## Introduction

This article discusses how analysing breastmilk for the presence of environmental chemicals bears the risk of undermining the confidence of mothers, parents and health professionals in breastfeeding and/or long term breastfeeding. In 2006, during the debate on REACH legislation, a report was presented in the European parliament entitled "Toxic inheritance – more than 300 pollutants in breastmilk" [1]. This publication issued by the NGOs "BUND" Germany and "Friends of the Earth Europe" was based on a translation of a German publication from 2005 which compiled data of biomonitoring for a number of substances in breastmilk from around the world. The focus was to alert parliamentarians to the presence of chemicals in humans, with special focus on vulnerable babies to guarantee more media attention. The presence of chemical residues in breastmilk is a shocking reality, yet breastfeeding is the natural way and optimal to feed infants and young children. Paediatricians and policy makers agree that the protection, promotion and support of breastfeeding are public health priorities. Today we know that breastfed children are healthier than infants receiving only some breast milk or none at all [2]. Therefore this article will present the biomonitoring results of breastmilk in a wider context linked to the ethical issue of communication to ensure consistency with public health policy.

## Breastmilk as a biomonitoring fluid

Breastmilk is often used in human biomonitoring to detect persistent residues of man-made chemicals accumulated in human bodies along the food chain. It is used as an indicator and as a monitoring instrument of fat soluble and persistent substances [3]. Breastmilk is often seen as an easy tool for biomonitoring because it is non invasive, though this ignores the fact that it may not be easy for the mother to deliver the needed amount, as pumping or expressing breastmilk can be problematic for some women. Information on chemicals in breastmilk may affect the mother and her social network in a way that undermines their confidence in the continuation of breastfeeding. Our association is regularly questioned by anxious parents or by health professionals after headlines on residues in breastmilk have been issued.

Today we can detect a wide range of foreign substances in breastmilk. The results presented are the mirror of chemical substances accumulated over the lifespan in the bodies of both men and women. The biomonitoring of breastmilk reveals residues of pesticides like DDT, Lindane, HCH and their breakdown products as well as chemicals like PCB, Bisphenol A, phthalates, synthetic perfumes (musks), flame retardants, sunscreen, perchlorate and chemical by-products like dioxins [1,4]. The fact that these substances are to be found in breast milk indicates that they are present in our bodies before conception and may have an effect on male or female reproductive cells; the chemicals are present in the womb thus affecting the direct environment of the fragile developing foetus. These substances can harm conception by affecting fertility or by having teratogenic effects on reproductive cells. This issue is now discussed in numerous scientific articles. Studies have shown that semen quality and semen counts deteriorate [5-7]. Male-mediated developmental toxicity is a concept becoming more widely known to researchers in this field [8-10]. The paternal impact on reproductive disorders and childhood cancers as a result of occupational exposure has been documented for e.g. pesticides or wood preservatives [11-13]. But this impact of residues remains less discussed amongst the wider public, whereas residues found in breastmilk are discussed or used for media headlines to grab attention. The attention given to breastmilk may be triggered by the fact that breastmilk, containing pollutants, is violating a basic social concept of 'purity' of this food and this unique relationship of a mother and baby.

Repeated biomonitoring of breastmilk or other body fluids provides the evidence base for strengthening legislation to phase out or reduce chemicals such as PCB and DDT. Effective legislation has had beneficial results: the levels of these chemicals measured as concentrations in our bodies are lower today than 10 or 20 years ago [4,14]. Comparing biomonitoring results between countries with and without legislation for reduced use of chemicals shows great differences.

## Breastfeeding as a unique food, containing live protective factors

"Breastfeeding is the natural way to feed infants and young children. Exclusive breastfeeding for the first six months of life ensures optimal growth, development and health. After that, breastfeeding, with appropriate complementary foods continues to contribute to the infant's and young child's nutrition development and health", as cited in the document: "Protection, promotion and support of breastfeeding in Europe: a Blueprint for action."[15]. This explains why breastmilk needs to be protected from environmental pollution and why paediatricians and policy makers agree that protection, promotion and support of breastfeeding are a public health priority [2,16]. Breastmilk contains an ample set of potent beneficial substances such as: Special long chain polyunsaturated fatty acids, lysozymes, secretory IgA, lactoferrin, lipase, prostaglandins, macrophages, cytokines, epidermal growth factor, relaxin, interferon, antioxidants, leucocytes, adiponectin and leptin to name only a few [17-19]. These substances are active in neural maturation, in building up the immune system, in gastrointestinal maturation, in immune defence and in setting and regulating body functions and metabolic processes. Some substances have antiviral, antiparasitic and antibacterial activity. It has been shown that infants fed on breastmilk substitutes such as infant formula are less healthy than breastfed infants. Many studies have proved that there is a dose dependent effect; the more and longer an infant is breastfed, the more beneficial is the effect on growth and healthy development. When infants are breastfed their immune system is better developed, the brain and neurological system can mature in an optimal way and the gut system develops better. Breastfed infants suffer less from respiratory diseases, diarrhoea, otitis media and urinary tract infections. They have optimal visual and intellectual development and a reduced risk of Diabetes type 1 and 2, allergic diseases, obesity, Crohn's disease, malignant lymphomas and cardiovascular disease in adulthood [2]. It can be concluded that breastfeeding strengthens the infant's developing immune system and decreases the incidence or severity of infectious diseases. Breastfeeding is also associated with a slightly enhanced performance on tests of cognitive development. Studies of cognitive development in relation to breastfeeding have all been performed in western environments with present levels of pollution. Based on the strong scientific evidence of the benefits of breastfeeding the American Academy of Pediatrics [2], WHO, UNICEF [16] and other organisations of health professionals recommend exclusive breastfeeding for the first six months of life and continued breastfeeding up to two years and beyond. The American Paediatric Association recommends in their latest policy document that: "Breastfeeding should be continued for at least the first year of life and beyond for as long as mutually desired by mother and child. There is no upper limit to the duration of breastfeeding and no evidence of psychologic or developmental harm from breastfeeding into the third year of life or longer"[2]. The WHO recommendation for optimal infant and young child feeding is: "The expert consultation recommends exclusive breastfeeding for six months, with introduction of complementary foods and continued breastfeeding thereafter for up to two years of age or beyond" [16]. The recommendation was adopted by all WHO member governments in 2002 at the World Health Assembly in the Global Strategy on Infant and Young Child Feeding [16]. This is a global public health recommendation and should be implemented at national level.

### The evidence base: Study results of perinatally exposed cohorts

Prenatal exposure to chemicals during pregnancy, when the vulnerability of the foetus is highest, may harm or impair the neurological and cognitive development of the unborn child and may have a negative impact on the immune system. Numerous cohort studies on infants born and living in exposed regions conclude that a negative impact on cognitive and neurological development is measurable for exposure in uterus. Findings from these cohort studies, which included biomonitoring and monitoring of breastfed status and duration of breastfeeding, may be unexpected for those looking only at the measurements and the figures of the residues in breastmilk and ignoring the bioactive factors contained in breastmilk. Breastmilk enhances immunologic development and gut maturation, it has antimicrobial and anti-inflammatory properties [20].

Boersma and Lanting showed that at six years of age cognitive development is affected by prenatal exposure to PCBs and dioxins [21]. Breastfed children however, when compared to formula fed children, had an advantage in terms of quality of movements, fluency and cognitive development tests at 18 and 42 months of age and at six years, despite a higher PCB exposure from breast milk. The conclusions of Boersma and Lanting were: "These data give evidence that prenatal exposure to PCBs do have subtle negative effects on neurological and cognitive development of the child up to school-age (....). Our studies showed evidence that breast feeding counteracts the adverse developmental effects of PCBs and dioxins" [21].

The results are supported by a study of Ribas-Fitó et al., who studied a birth cohort of 92 mother-infant pairs highly exposed to organochlorine compounds. The researchers published their findings in 2003 in Pediatrics and their conclusion stated: "Prenatal exposure to p,p'DDE was associated with a delay in mental and psychomotor development at 13 months. Long-term breastfeeding was found to be beneficial to neurodevelopment, potentially counterbalancing the impact of exposure to these chemicals through breast-milk" [22].

In a review Feeley and Brouwer concluded that: "Little if any adverse health effects have been associated with breast-feeding. In fact, the beneficial effect from breast-feeding on neurological measures has been observed" [23].

The positive results of breastfeeding are also found in an exploratory study by Vreugdenhil et al. who suggest that prenatal exposure to environmental levels of PCBs and related compounds delays mechanisms in the central nervous system that evaluate and process relevant stimuli, whereas breastfeeding accelerates these mechanisms [24].

In recent studies the issue of cognitive development and breastfeeding has been further addressed. In a study from Pediatrics published in 2006 by Charnley and Kimbrough the researchers state that: "Breast-fed infants have higher exposures than formula-fed infants, but studies consistently find that breast-fed infants perform better on developmental neurologic tests than their formula-fed counterparts, supporting the well-recognized benefits of breast feeding" [25]. The benefits of breastfeeding despite exposure to higher levels of chemicals were also found in another recent study published in Pediatrics by Eskenazi et al, who concluded that: "Prenatal exposure to DDT, and to a lesser extent DDE, was associated with neurodevelopmental delays during early childhood, although breastfeeding was found to be beneficial even among women with high levels of exposure" [26].

These findings all show what happens with breastfed infants born into our present polluted environment. However, because of the lack of a control group living in an unexposed environment, it is not possible to assess the total positive health and development impact of breastfeeding.

## Communication of results

In the media breastmilk, when used as a biomonitoring tool for analysing the human body burden, is often portrayed as being contaminated with persistent organic pollutants [1]. Sensational messages about contaminants in breastmilk undermine the value of breastfeeding and the confidence of parents and health care professionals in breastfeeding and long term breastfeeding. Perceptions created by communication of the findings of residues detected in breastmilk place the focus on breastmilk, thereby indirectly blaming the breastfeeding mother. Attention is drawn away from the pollutants which create this situation and from the industries that produce, use or release the chemicals and thereby create environmental pollution. Attention is deflected from the fact that these persistent chemicals accumulate in the food chain and have adverse effects that are more or less documented on all species high up on the food chain, including human beings. Nevertheless NGOs have legitimate normative and political reasons to expose this, in trying to galvanise public pressure for political action to prevent, reduce and eliminate the sources of contaminating chemicals. In current messages communicating the detection of residues in breastmilk, the whole situational context is overlooked: "polluted breastmilk" is the main focus and avoidance of breastfeeding is seen as a possible option. It is forgotten that breastmilk is an indicator of the bioaccumulation of persistent chemical substances in both men and women. The questioning of female body fluids, with "white" and "pure" milk portrayed as being polluted and "impure", leads emotions into ancient fears. Insensitive communication of biomonitoring results focussing only on the presence of substances that should not be in breastmilk and their measured values undermines the confidence of mothers, fathers, and health professionals in breastfeeding. It is very important that communication on residues detected in breastmilk during biomonitoring is seen as an indicator of the body burden of all human beings and that the focus is not only directed towards "contaminated breastmilk". The results should be an exhortation to political action for strong legislation on the production, release, and use of these chemicals detected in breastmilk. The results should not be used to undermine confidence in breastmilk as the optimal food for infants and young children. The existence of chemical residues in breastmilk is not a reason for limiting breastfeeding. On the contrary those findings are one of the reasons to breastfeed because breastmilk contains substances that help the child develop a stronger immune system and optimal neural and brain maturation. Thus breastfeeding can help limit the damage caused by foetal exposure to these chemicals.

### Examples of good communication messages

For persons involved in human biomonitoring of breastmilk it is paramount to protect breastmilk and breastfeeding as the best possible nutrition for growth and healthy development of infants and young children [27,28]. This can be done by elucidating a comprehensive picture of exposure, as explained above. To include lactation specialists e.g. lactation consultants and NGOs working on protection and support of breastfeeding in planning and communication efforts could also be a valuable tool. Agencies and NGOs active in biomonitoring of breastmilk are becoming aware of this situation and of the need for a change in their communication methods [29].

*In May 2005, WHO and UNEP entered into a Memorandum of Agreement for the coordination of human milk surveys for the purpose of the Stockholm Convention*.

*"At the same time, evidence for the health advantages of breastfeeding and scientific evidence to support breastfeeding has continued to increase*.

• *WHO can now say with full confidence that breastfeeding reduces child mortality and has health benefits that extend into adulthood*.

On a population basis, exclusive breastfeeding for six months is the recommended feeding mode for the vast majority of infants, followed by continued breastfeeding with appropriate complementary foods for up to two years or beyond."

In 2005, the World Health Organisation (WHO) issued the 4th WHO coordinated survey of human milk for persistent organic pollutants with the following message: "In all cases human milk will be promoted as the naturally superior food for infants (...) This survey being consistent with the promotion of human milk as the optimal food for infants could provide the ideal basis for possible source-directed measures to ultimately reduce levels of POPs in human milk" [3]. In the revised protocol of 2007 WHO, now in cooperation with UNEP (United Nations Environment Programme) goes beyond this by extending the messages about breastfeeding [3,30,31]; "WHO can now say with full confidence that breastfeeding reduces child mortality and has health benefits that extend into adulthood. On a population basis, exclusive breastfeeding for six months is the recommended feeding mode for the vast majority of infants, followed by continued breastfeeding with appropriate complementary foods for up to two years or beyond" [30]. WHO and UNEP have now developed an annex to their Protocol for Collection, Handling and Analysis of Samples at the Country Level named "The value of breastfeeding" [30], which contains following message: "Breastfeeding is the ideal way to feed infants; its benefits go far beyond sound nutrition, and children should not needlessly be deprived of it" [30].

In this protocol WHO has even gone beyond the messages quoted above by not only targeting organisers of biomonitoring of breastmilk and field workers in contact with mothers, but by also including communication addressed directly to mothers. They have developed a prenatal information sheet on breastfeeding for mothers selected to be included in their survey.

Messages such as those above, including scientific evidence both promote and protect breastfeeding whenever breastmilk is collected, sampled and analysed for biomonitoring.

Awareness about similar caution in communication is being discussed when publishing levels of chemicals in fish versus the positive health effects of consuming fish, or residues of pesticides in fruit and vegetables while promoting a recommended amount of fruit or vegetables a day.

## Conclusion

Breastfeeding rates in Europe are rising but they are still far away from the recommendation of the World Health Organisation, as shown in Cattaneo [15]: it is therefore important that messages about biomonitoring results do not harm or undermine public health messages for the protection, promotion and support of breastfeeding. On the other hand, breastmilk and breastfeeding should be protected against harmful substances and harmful messages. The communication efforts by WHO to ensure policy consistency provide a good example and should be implemented whenever breastmilk is analysed.

## Competing interests

The authors declare that they have no competing interests.
